# Exploring the Impact of Cigarette Smoke Extracts on Vitamin B_12_: Insights into the Transformation of Methylcobalamin and Hydroxycobalamin to Cyanocobalamin through *In Vitro* Evaluation

**DOI:** 10.1155/2024/8827402

**Published:** 2024-04-18

**Authors:** Mazhar Salim Al Zoubi, Mus'aab A. Al-Oun, Fatima Yacoub Abusahyoun, Manal Issam Abualarja, Asmaa Al Smadi, Bahaa Al-Trad, Sura A. Awadin, Khalid Al-Batayneh, Mai Elaarag, Raed M. Al-Zoubi

**Affiliations:** ^1^Department of Basic Medical Sciences, Faculty of Medicine, Yarmouk University, Irbid 211-63, Jordan; ^2^Department of Biological Sciences, Faculty of Sciences, Yarmouk University, Irbid 211-63, Jordan; ^3^Surgical Research Section, Department of Surgery, Hamad Medical Corporation, Doha, Qatar; ^4^Department of Chemistry, Jordan University of Science and Technology, Irbid 22110, Jordan; ^5^Department of Biomedical Sciences, College of Health Sciences, Qatar University, Doha 2713, Qatar

## Abstract

Vitamin B_12_ (cobalamin) is a water-soluble molecule required for the proper functioning of metabolism, blood and DNA synthesis, and neurological development. Vitamin B_12_ exists in several forms: methylcobalamin (MeCbl), adenosylcobalamin (AdoCbl), hydroxycobalamin (OHCbl), and cyanocobalamin (CNCbl). This study aimed to evaluate the effect of cigarette smoke on the chemical structure of methylcobalamin and hydroxycobalamin forms of vitamin B_12_. MeCbl and OHCbl were markedly affected by exposure to cigarette smoke. The resemblance of the Rt between MeCbl and OHCbl and CNCbl indicates that exposure to cigarette smoke extracts chemically alters MeCbl and OHCbl to CNCbl, warranting *in vivo* research investigations.

## 1. Introduction

Vitamin B_12_ (cobalamin) is a water-soluble vitamin that plays an important role in certain metabolic processes [1, 2]. The main supply of cobalamin comes from animal resources such as liver, kidney, meat, egg, and milk derivatives. The highest level of cobalamin comes from fish, and there is a small level that is produced by some kind of intestinal macrobiotic [3]. In the last few decades, there was a lot of debate about cobalamin affectivity and deficiency. It was found that nearly 6% of Western people at the age of 60 had a low level of cobalamin serum and around 20% have shown marginal cobalamin status [4]. This subtle deficiency may be responsible for cognitive function and may cause dementia in older people due to poor food habits and poor absorption which may lead to a low cobalamin concentration [5, 6]. Vitamin B_12_ has many vital roles such as the synthesis of DNA, some neurological functions, and the metabolism of proteins and carbohydrates [7]. Furthermore, it has a role in myelin synthesis and erythropoiesis [8, 9]. The most significant symptoms related to vitamin B_12_ deficiency are fatigue, memory impairment, skin pallor, glossarist, and severe hematological and neurological disorders [10–14].

**Figure 1:**
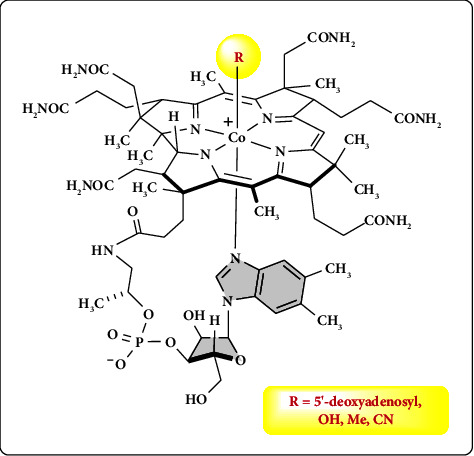
Vitamin B_12_ chemical structure and forms. *R* is bound to cobalt from the *β*-face.

Vitamin B_12_ has a complex structure that contains a corrin ring that includes four pyrrole rings and a central cobalt ion that is attached to four nitrogen atoms as illustrated in Figure 1. In addition, a dimethyl benzimidazole group and variable *R* group are located below and above the plane of the corrin ring, respectively [9, 15–18].

Vitamin B_12_ exists in several forms; one of them being methylcobalamin (MeCbl), it is an active form that is necessary to maintain the nervous system and the most efficient form that is consumed by neurons and cells. MeCbl form, considered a cofactor for methionine synthase in the methionine synthesis process is used in the treatment of vitamin B_12_ deficiency and Alzheimer's disease [19, 20]. Another active analog of vitamin B_12_ is adenosylcobalamin (AdoCbl), a very sensitive derivative to light, which is known as a coenzyme for methyl malonyl-CoA mutase [21–23]. Cyanocobalamin (CNCbl), an inactive form of vitamin B_12_, is described as an element of antipernicious anemia, exists in trace amounts in food and is eliminated quickly in the urine [24]. CNCbl is not a coenzyme and is mostly used as a supplement. It is partially transformed into active forms upon being absorbed in the intestine [21–23, 25, 26]. Hydroxycobalamin (OHCbl) is a unique form that is converted into its active form and has a relatively long shelf life with minimal side effects. Notably, OHCbl is one of the effective therapies used in cases of cyanide poisoning [21, 25].

The absorption process of vitamin B_12_ involves the ingestion of vitamin B_12_ (cobalamin); it will bind first to haptocorrin within the salivary glands and then will undergo a proteolytic cleavage via the stomach to the duodenum where pancreatic proteases break down haptocorrin proteins and release vitamins. The intrinsic factor attaches to cobalamin to form a complex that is actively absorbed through cubilin receptors at the ileum level. In the enterocytes, the cobalamin is released from the intrinsic factor and exported to the circulation, where it binds with transcobalamin. Enterohepatic circulation releases cobalamin into the bile associated with haptocorrin. This cobalamin may be reabsorbed into the ileum if there is an intrinsic factor [27]. The two distinct metabolic cascades have been established in the two active forms of vitamin B_12_ (methylcobalamin and adenosylcobalamin (AdoCbl)) [28–30]. AdoCbl form is stored in the cellular tissues, particularly in the mitochondria. Other forms of vitamin B_12_ are found in the cytosol, blood and some body fluids [31].

Various factors have been suggested to affect the structural integrity and conformation of vitamin B_12_ such as heat, light, and microwaves, and to lead to vitamin B_12_ deficiency [32–36]. Likewise, cigarette smoking has been proposed to be a causative agent of vitamin B_12_ deficiency by possibly converting the active forms of vitamin B_12_ into cyanocobalamin. This inactive form is, in turn, excreted with urine [24]. The mechanism of this conversion and inactivation of vitamin B_12_ is enigmatic; however, certain ingredients of the smoke such as organic nitrates, nitrous oxides, cyanate, and isocyanates could be responsible, warranting critical research assessments [37]. Therefore, in the current study, we aimed to evaluate the *in vitro* effect of cigarette smoke on the structure and integrity of methylcobalamin and hydroxycobalamin forms of vitamin B_12_.

## 2. Materials and Methods

**Figure 2:**
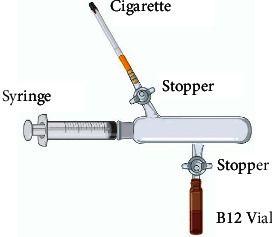
Cigarette smoke extraction apparatus which consists of a syringe with equal volume collection champer with two valved exits for CSE collection and cobalamin vial connection.

Three different forms of cobalamin were used in this experiment (MeCbl, OHCbl, and CNCbl) where the concentrations were 1000 *μ*g/mL. Vitamin B_12_ forms were obtained from the local pharmaceutical market (Jordan pharmaceutical manufacturing, Rotexmedica, and Panbiotic laboratories). Cigarette smoke was extracted by a homemade device (Figure 2) and mixed with the methylcobalamin and hydroxycobalamin solutions at different concentrations (i.e., 5 cigarettes, 10 cigarettes, and 20 cigarettes) in triplicates. The mixing process was performed under dark conditions due to the sensitivity of vitamin B_12_ to light. Samples were examined on auto-sampler high-performance liquid chromatography using Thermo Scientific, Dionex UltiMate-3000 Series. C8-column Phenosphere 5 *μ*m with 250 *∗* 4.6 mm internal diameter from (Phenomenex, USA) and variable wavelength detectors (VWD-3100 and VWD-3400RS) were used. Two mobile phases were used, and gradient elution was performed with 0.025% (w/v) trifluoroacetic acid (TFA) in water (mobile phase A) and pure acetonitrile (mobile phase B) at a flow rate of 1.0 mL/min.

### 2.1. Treatment by Cigarette Smoke Extracts

Each form of cobalamin (OHCbl, MeCbl, and CNCbl) was divided into three groups (5.0 mL each (1000 *μ*g/mL)) and mixed with cigarette smoke extracts (5 cigarettes, 10 cigarettes, and 20 cigarettes).

### 2.2. Treatment by Potassium Cyanide (KCN)

An additional experiment was performed to confirm the role of cyanide (CN) in cigarette contents by the conversion of vitamin B_12_ forms to CNCbl. Briefly, 0.1 mM of KCN was prepared and mixed with OHCbl and measured by High-Performance Liquid Chromatography (HPLC).

### 2.3. High-Performance Liquid Chromatography (HPLC)

The samples were analyzed by using the HPLC auto-sampler (Thermo Scientific) with variable wavelength detectors (VWD-3100 and VWD-3400RS) (Germany). Gradient elution was performed with 0.025% (w/v) trifluoroacetic acid (TFA) in water (mobile phase A) and pure acetonitrile (mobile phase B) at a flow rate of 1.0 mL/min. The total run time was 18.0 minutes for each sample. C8-column Phenosphere 5 *μ*m with 250 *∗* 4.6 mm internal diameter from (Phenomenex, USA) was used in the study.

## 3. Results

### 3.1. HPLC Analysis

**Figure 3:**
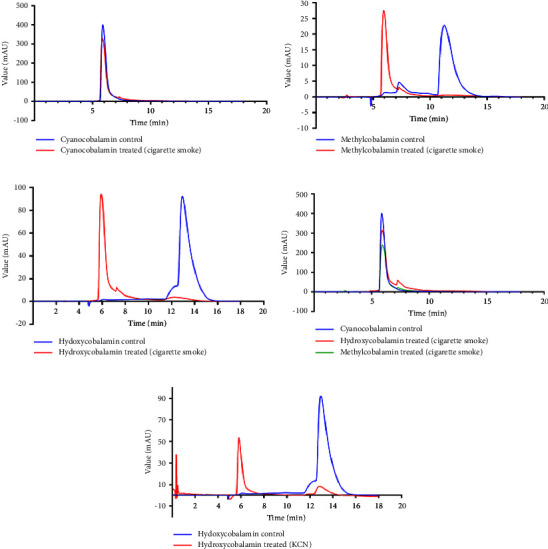
HPLC chromatogram of both control and CSE-treated samples (5 cigarettes); blue: control, red: treated with cigarette smoke extracts: (a) control and treated samples of cyanocobalamin; (b) control and treated samples of methylcobalamin; (c) control and treated samples of hydroxycobalamin; (d) control cyanocobalamin with treated samples of hydroxycobalamin and methylcobalamin; (e) KCN-treated hydroxycobalamin.

The HPLC results showed no impact of cigarette smoke treatment on cyanocobalamin, giving the same peak and retention time (Rt) (6 min) as shown in Figure 3(a). On the other hand, the HPLC results showed a drastic change in the Rt time of both methylcobalamin and hydroxycobalamin after exposure to cigarette smoke extracts as shown in Figures 3(b) and 3(c), respectively. The chromatogram clearly showed a change in the Rt of methylcobalamin peak from 11.5 mins en route to 6.0 mins for both control and treated samples, respectively. For hydroxycobalamin, the Rt also shifted from 13.0 mins to 6.0 mins for both control and treated samples, respectively, as shown in Figure 3(c).

The inherent overlapping in Rt of both treated OHCbl and MeCbl with the untreated CNCbl implies the chemical conversion of OHCbl and MeCbl to CNCbl after treatment with cigarette smoke extracts (Figure 3(d)).

To confirm the chemical change in both hydroxycobalamin and methylcobalamin-treated samples and also to investigate further the role of cyanide (CN) extracted from the cigarette smoke in the conversion of OHCbl and MeCbl to CNCbl, we performed a direct mix between hydroxycobalamin and KCN solution at a concentration of 0.1 mM. It provided a new peak, which is matched perfectly in Rt with the peak of treated OHCbl with cigarette smoke extracts as shown in Figure 3(e). Interestingly, the results showed that the different doses of cigarette smoke extracts (5, 10, and 20 cigarettes) have the same effect on vitamin B_12_ forms.

## 4. Discussion

Vitamin B_12_ deficiency is a worldwide health concern and plays a vital role in many metabolic pathways in the human body [38–42]. Many pieces of evidence in the literature suggest that the structure of vitamin B_12_ is affected by several factors such as heat and UV light [32, 43]. Other studies reported an association between vitamin B_12_ deficiency and smoking. Accordingly, we assumed in this study that the exposure effect of vitamin B_12_ forms to cigarette smoke extracts might alter the chemical structure and therefore, lose their benefits as a vitamin in our body. According to the structure of vitamin B_12_, we believe that the exposure of vitamin B_12_ forms to cigarette smoke extracts will be able to exchange the perpendicular *R* group, giving one of the existing forms or a new and inactive form. The three most common forms (OHCbl, MeCbl, and CNCbl) were used in this study. The experiments were conducted by treating OHCbl, MeCbl, and CNCbl with cigarette smoke extracts. Only one of these three forms of vitamin B_12_ is known to be inactive, the CNCbl form, which is known to be excreted by urine from the body.

The HPLC chromatogram of the cigarette-smoke-treated MeCbl showed a significant shift in the Rt from 12 mins to 6 mins resembling the Rt of the CNCbl. No difference in Rt for CNCbl (control) and cigarette-smoke-treated CNCbl (treated). The varying doses of cigarette smoke extracts have an identical impact on the forms of vitamin B_12_. These results are in line with a previous study that demonstrated that urine B_12_ excretion was raised in smokers and a relatively low serum B_12_ concentration [44]. It is worth noting, as numerous other published manuscripts have indicated, that it is challenging to ensure that upon burning the same quantity of cigarettes, even of the same brand, consistently contain identical quantities and compositions of cigarette extracts.

In several studies, smoking is related to the reduced vitamin B_12_ concentration in serum and the increased vitamin B_12_ secretion through the urine. For instance, a study reported a significant reduction in B_12_ concentration in the serum in the smokers' cohort compared to the nonsmokers' control sample. The B_12_ concentration in serum was 444 *μ*g/ml and 472 *μ*g/ml for both smokers and nonsmokers patients, respectively [43]. Additionally, the authors reported a significant increase in the concentration of B12 in urine samples (81.2 m *μ*g/24 hours) for the smokers' cohort compared to 60.3 m *μ*g/24 hours for nonsmokers' control. Overall, cigarette smoking will possibly decrease vitamin B12 concentration in serum by increasing its excretion in urine [44]. Singh reported a study that enrolled 300 males, 150 among them, were chronic cigarette smokers who have been smoking for more than 20 years and the other 150 were nonsmokers recruited as a control group. Their results showed that the concentration of vitamin B12 in the smokers' group was 346 pg/ml, whereas the concentration of B12 in nonsmokers was 481 pg/ml. The findings of these studies are steady with our results that hydroxycobalamin and methylcobalamin can be converted to cyanocobalamin by altering the *R* group in cobalamin to be CN and then excreted B12 out of the body leading to a decline in B12 concentration in the serum [45]. In addition, another study recruited 33 pregnant women between 16 and 22 weeks of gestation, among them, 19 patients were smokers and 14 were nonsmokers. Their findings reported a lower concentration of vitamin B12 in the smokers' group compared to the nonsmokers' control group [46]. Pagán et al. published a study on the effect of smoking on vitamin B12, 285 women in mid-pregnancy were enrolled and showed a significant difference in vitamin B12 concentration between the smokers' group and the nonsmokers' group [47]. A systematic review included 13 studies with a total of 8661 patients concluded that low levels of vitamin B12 in smokers compared to nonsmokers were found in 8 out of 13 studies [48]. Collectively, the previously published reports and their findings support our results of smoking's effect on vitamin B12 structure and conversion of vitamin B12 to CNCbl and therefore, led to a lower vitamin B12 concentration in blood. Moreover, CNCbl was not recommended for smokers due to a possible alteration in the metabolism of CNCbl and an increase in excretion [44]. Many studies and research have shown the superior tissue retention rates of Cbl after the OHCbl supplement was taken instead of the cyano-Cbl (CNCbl) which led to a rise in the urinary secretion of CNCbl [49–57].

The results of the current study demonstrated the possible impact of cigarette smoke extracts on the structure of cobalamin formulas *in vitro*, however, this proof principle approach requires further in vivo experiments to provide us with the exact effect of smoking on vitamin B12 status in humans. Despite the previous reports about the association between smoking and vitamin B12 deficiency, more updated and well-designed experiments are required to elucidate this correlation since some of these reports were conducted more than 40 years ago. For instance, the case-control study with follow-up measures will be a good starting-up approach to finding a correlation between cigarette smoking and vitamin B12 levels. To the best of our knowledge, this is the first study that reports an alteration in vitamin B12 structure postcigarette smoke treatment. Further investigation into the effect of thiocyanate in vitamin B12 forms is needed.

## 5. Conclusion

The shifting in the Rt time of treated MeCbl and OHCbl forms of vitamin B_12_ indicates that exposure to cigarette smoke induces a chemical conversion to CNCbl. This is likely correlated with decreased levels of vitamin B_12_ in smokers and its loss after supplement administration. Thus, smokers diagnosed with vitamin B_12_ deficiency require primary healthcare and medical consultation. Further, *in vivo* studies are recommended to verify our results.

## Data Availability

The data that supports the findings in this study are available from the corresponding authors upon reasonable request.

## Additional Points

*Highlights*. (i) MeCbl and OHCbl were markedly affected by exposure to cigarette smoke extracts. (ii) Exposure to cigarette smoke extracts chemically altered MeCbl and OHCbl to CNCbl. (iii) This study suggests that cigarette smoking may cause a deficiency in vitamin B12 due to the formation of urine excretable form of vitamin B12 (CNCbl).
